# Three-Dimensional Numerical Model of Cell Morphology during Migration in Multi-Signaling Substrates

**DOI:** 10.1371/journal.pone.0122094

**Published:** 2015-03-30

**Authors:** Seyed Jamaleddin Mousavi, Mohamed Hamdy Doweidar

**Affiliations:** 1 Group of Structural Mechanics and Materials Modeling (GEMM), Aragón Institute of Engineering Research (I3A), University of Zaragoza, Zaragoza, Spain; 2 Mechanical Engineering Department, School of Engineering and Architecture (EINA), University of Zaragoza, Zaragoza, Spain; 3 Centro de Investigación Biomédica en Red en Bioingeniería, Biomateriales y Nanomedicina (CIBER-BBN), Zaragoza, Spain; University of Zurich, SWITZERLAND

## Abstract

Cell Migration associated with cell shape changes are of central importance in many biological processes ranging from morphogenesis to metastatic cancer cells. Cell movement is a result of cyclic changes of cell morphology due to effective forces on cell body, leading to periodic fluctuations of the cell length and cell membrane area. It is well-known that the cell can be guided by different effective stimuli such as mechanotaxis, thermotaxis, chemotaxis and/or electrotaxis. Regulation of intracellular mechanics and cell’s physical interaction with its substrate rely on control of cell shape during cell migration. In this notion, it is essential to understand how each natural or external stimulus may affect the cell behavior. Therefore, a three-dimensional (3D) computational model is here developed to analyze a free mode of cell shape changes during migration in a multi-signaling micro-environment. This model is based on previous models that are presented by the same authors to study cell migration with a constant spherical cell shape in a multi-signaling substrates and mechanotaxis effect on cell morphology. Using the finite element discrete methodology, the cell is represented by a group of finite elements. The cell motion is modeled by equilibrium of effective forces on cell body such as traction, protrusion, electrostatic and drag forces, where the cell traction force is a function of the cell internal deformations. To study cell behavior in the presence of different stimuli, the model has been employed in different numerical cases. Our findings, which are qualitatively consistent with well-known related experimental observations, indicate that adding a new stimulus to the cell substrate pushes the cell to migrate more directionally in more elongated form towards the more effective stimuli. For instance, the presence of thermotaxis, chemotaxis and electrotaxis can further move the cell centroid towards the corresponding stimulus, respectively, diminishing the mechanotaxis effect. Besides, the stronger stimulus imposes a greater cell elongation and more cell membrane area. The present model not only provides new insights into cell morphology in a multi-signaling micro-environment but also enables us to investigate in more precise way the cell migration in the presence of different stimuli.

## Introduction

Cell shape change during cell migration is a key factor in many biological processes such as embryonic development [1–3], wound healing [4–6] and cancer spread [7–9]. For instance, during embryogenesis the head-to-tail body axis of vertebrates elongates by convergent extension of tissues in which cells intercalate transversely between each other to form narrower and long body [1]. Besides, after an injury in the cornea, the healing process is followed by epithelial shape changes during cell migration. Epithelials near the wound bed change their shape to cover the defect without leaving intercellular gaps. The greatest cellular morphological alterations are observed around the wound edges. Remote cells from wounded regions migrate towards the wound center and are elongated during migration in the migration direction, increasing their membrane area. As the healing proceeds, the cell original pattern is changed which is recovered after wound healing [4]. Invasion of cancerous cells into surrounding tissue needs their migration which is guided by protrusive activity of the cell membrane, their attachment to the extracellular matrix and alteration of their micro-environment architecture [9]. Many attempts have been made to explain cell shape changes associated with directed cell migration, but the mechanism behind it is still not well understood. However, it is well-known that cell migration is fulfilled via successive changes of the cell shape. It is incorporated by a cyclic progress during which a cell extends its leading edge, forms new adhesions at the front, contracts its cytoskeleton (CSK) and releases old adhesions at the rear [10, 11]. A key factor of the developmental cell morphology is the ability of a cell to respond to directional stimuli driving the cell body. Several factors are believed to control cell shape changes and cell migration including intrinsic cue such as mechanotaxis or extrinsic stimuli such as chemotaxis, thermotaxis and electrotaxis.

For the first time Lo et al. [12] demonstrated that cell movement can be guided by purely physical interactions at the cell-substrate interface. After, investigations of Ehrbar et al. [13] illustrated that cell behavior strongly depends on its substrate stiffness. During cell migration in consequence of mechanotaxis, amoeboid movement causes frequent changes in cell shape due to the extension of protrusions in the cell front [14, 15], which is often termed pseudopods or lamellipods, and retraction of cell rear. Therefore, during this process, protrusions develop different cell shapes that are crucial for determination of the polarization direction, trajectory, traction forces and cell speed.

In addition to mechanotaxis, gradient of chemical substance or temperature in the substrate gives rise to chemotactic [16, 17] or thermotactic [18, 19] cell shape changes during migration, respectively. Existent chemical and thermal gradients in the substrate regulate the direction of pseudopods in such a way that the cell migrates in the direction of the most effective cues [19, 20]. However, it is actually myosin-based traction force (a mechanotactic tool) that provides the force driving the cell body forward [12, 21]. Recently, a majority of authors have experimentally considered cell movement in the presence of chemotactic cue [17, 20] demonstrating that a shallow chemoattractant gradient guides the cell in the direction of imposed chemical gradient such that the extended pseudopods and cell elongation are turned in the direction of the gradient [20]. In contrast, some cells such as human trophoblasts subjected to oxygen and thermal gradients do not migrate in response to oxygen gradient (a chemotactic cue) but they elongate and migrate in response to thermal gradients of even less than 1°C towards the warmer locations [19]. However, there are some other cases such as burn traumas, influenza or some wild cell types that cell may migrate towards the lower temperature, away from warm regions [22].

Recent *in vitro* studies have demonstrated that the presence of endogenous or exogenous electrotaxis is another factor for controlling cell morphology and guiding cell migration [23–28]. Influence of endogenous Electric Fields (EFs) on cell response was first studied by Verworn [29]. Experimental evidences reveal important role of endogenous electrotaxis in directing cell migration during wound healing process during which the cell undergoes crucial shape changes [30, 31]. In the past few years, there has also been a growing interest in the effects of an exogenous EF on cells in culture, postulating that calcium ion, Ca^2+^, is involved in electrotactic cell response [27, 32–37]. A cell in natural state have negative potential that exposing it to an exogenous direct current EF (dcEF) causes extracellular Ca^2+^ influx into intracellular through calcium gates on the cell membrane. Subsequently, in steady state, depending on intracellular content of Ca^2+^, a typical cell may be charged negatively or positively [38]. This is the reason that many cells such as fish and human keratinocytes, human corneal epithelials and dictyostelium are attracted by the cathode [26, 39–42] while some others migrate towards the anode, e.g. lens epithelial and vascular endothelial cells [39, 43]. Although, experiments of Grahn et al. [44] demonstrate that human dermal melanocyte is unexcitable by dcEFs, it may occur due to its higher EF threshold [36].

To better understand how each natural biological cue or external stimulus influences the cell behavior, several kinds of mathematical and computational models have been developed [17, 45–54]. Some of these models commonly simulate the effect of only one effective cue on cell migration [50, 52, 55] while some others at most deal with mechanotactic and chemotactic cues, simultaneously [17, 51]. There are several energy based mathematical models considering the effect of substrate rigidity on cell shape changes [52, 56]. They assumed that the cell morphology is changed by the energy stored in cell-substrate system, thus, minimization of the total free energy of the system defines the final cell configuration [52]. 2D model presented by Neilson et al. [51] simulates eukaryotic cell morphology during cell migration in presence of chemotaxis by employing a system of non-linear reaction-diffusion equations. The cell boundary is characterized using an arbitrary Lagrangian-Eulerian surface finite element method. The main advantage of their model is prediction of the cell behavior with and without chemotactic effect although it has two key objections: (i) the cell movement is totally random in absence of chemotactic stimulus, missing mechano-sensing process; (ii) the study of the cell configurations is limited to elliptical modes. In addition, numerical model presented by Han et al. [49] predicts the spatiotemporal dynamics of cell behavior in presence of mechanical and chemical cues on 2D substrates. Considering constant cell shape, they assume that the formation of a new adhesion regulates the reactivation of the assembly of fiber stress within a cell and defines the spatial distribution of traction forces. Their findings indicates that the strain energy is produced by the traction forces which arise due to a cyclic relationship between the formation of a new adhesion in the front and the release of old adhesion at the rear.

Altogether, although, available models provide significant insights about cell behavior, they include several main drawbacks: (i) most of the present models incorporate signals received by the cell with mechanics of actin polymerization, myosin contraction and adhesion dynamics but do not deal with the traction forces exerted by the cell during cell movement [57–60]; (ii) some of available models simply simulate cell migration with constant cell configuration [57, 61]; (iii) models considering cell morphology only concentrate on the dynamics of cellular shapes which are not easily applicable for temporal and spatial investigation of cell shape changes coupled with cell movement [52, 62–65]; (iv) models predicting cell morphology are restricted to a few rigid cellular configurations [52, 62]; (v) some of existent models overlook mechanotactic process of cell migration [17, 50, 51] which is inseparable from cell-matrix interaction [12]. Apart from this shortages, most of the models dealing with cell migration and cell shape changes are developed in 2D [17, 52, 55, 57–60] that according to the comprehensive experimental investigations of Hakkinen et al. [63], in many concepts cell behavior, particularly as for cell morphology, on 2D substrates strongly differs from that within 3D substrates. However in many viewpoints, 2D models improve our notions on cell motility and cellular configuration. Above all shortcomings mentioned before, to our knowledge, there is no comprehensive model to investigate cell shapes changes during cell-matrix interactions within multi-signaling environments (mechano-chemo-thermo-electrotaxis).

We have previously developed a 3D numerical model of cell migration within a 3D multi-signaling matrix with constant cell configuration [66, 67]. In addition, a novel mechanotactic 3D model of cell morphology is recently presented by the same authors [68]. The objective of the present work is to extend previously presented models [66–68] to investigate cell shape changes during cell migration in a 3D multi-signaling micro-environment. The model takes into account the fundamental feature of cell shape changes associated in cell migration in consequence of cell-matrix interaction. It relies on equilibrium of forces acting on cell body which is able to predict key spatial and temporal features of cell such as cell shape changes accompanied with migration, traction force exerted by the cell and cell velocity in the presence of multiple stimuli. Some of the results match with findings of experimental studies while some others provide new insights for performing more efficient experimental investigations.

## Model description

### Transmission of cell internal stresses to the substrate

Recent investigations have demonstrated that active (actin filaments and AM machinery) and passive (microtubules and cell membrane) cellular elements play a key role in generating the cell contractile stress which is transmitted to the substrate through integrins. The former, which generates active cell stress, basically depends on the minimum, *ϵ*_min_, and maximum, *ϵ*_max_, internal strains, which is zero outside of *ϵ*_max_-*ϵ*_min_ range, while the latter, which generates passive cell stress, is directly proportional to stiffness of passive cellular elements and internal strains. Therefore, the mean contractile stress arisen due to incorporation of the active and passive cellular elements can be presented by [66–69]

$$\begin{array}{c} \sigma =\left\{\begin{array}{cc} K_{\text{pas}}\epsilon_{\text{cell}} & \epsilon_{\text{cell}}<\epsilon_{\text{min}}\;\text{or}\;\epsilon_{\text{cell}}>\epsilon_{\text{max}}\\ \frac{K_{\text{act}}\sigma_{\text{max}}\left(\epsilon_{\text{min}}-\epsilon_{\text{cell}}\right)}{K_{\text{act}}\epsilon_{\text{min}}-\sigma_{\text{max}}}+K_{\text{pas}}\epsilon_{\text{cell}} & \epsilon_{\text{min}}\leq \epsilon_{\text{cell}}\leq \tilde{\epsilon }\\ \frac{K_{\text{act}}\sigma_{\text{max}}\left(\epsilon_{\text{max}}-\epsilon_{\text{cell}}\right)}{K_{\text{act}}\epsilon_{\text{max}}-\sigma_{\text{max}}}+K_{\text{pas}}\epsilon_{\text{cell}} & \tilde{\epsilon }\leq \epsilon_{\text{cell}}\leq \epsilon_{\text{max}} \end{array}\right. \end{array}$$ (1)

where *K*_pas_, *K*_act_, *ϵ*_cell_ and *σ*_max_ represent the stiffness of the passive and active cellular elements, the internal strain of the cell and the maximum contractile stress exerted by the actin-myosin machinery, respectively, while $\tilde{\epsilon }=\sigma_{\text{max}}/K_{\text{act}}$.

### Effective mechanical forces

A cell extends protrusions in leading edges in the direction of migration and adheres to its substrate pulling itself forward in direction of the most effective signal. The cell membrane area is as tiny as to produce strong traction force due to cell internal stress, consequently, adhesion is thought to compensate this shortage by providing the sufficient traction required for efficient cell translocation [3]. The equilibrium of forces exerted on the cell body should be satisfied by cell migration and cell shape changes [70, 71]. In the meantime, two main mechanical forces act on a cell body: traction force and drag force. The former is exerted due to the contraction of the actin-myosin apparatus which is proportional to the stress transmitted by the cell to the ECM by means of integrins and adhesion. Representing the cell by a connected group of finite elements, the nodal traction force exerted by the cell to the surrounding substrate at each finite element node of the cell membrane can be expressed as [69]

$$\begin{array}{c} F_{i}^{\text{trac}}=\sigma_{i}S\left(t\right)\zeta e_{i} \end{array}$$ (2)

where *σ*_*i*_ is the cell internal stress in *i*th node of the cell membrane and **e**_*i*_ represents a unit vector passing from the *i*th node of the cell membrane towards the cell centroid. *S*(*t*) is the cell membrane area which varies with time. During cell migration, it is assumed that the cell volume is constant [72–74], however the cell shape and cell membrane area change. *ζ* is the adhesivity which is a dimensionless parameter proportional to the binding constant of the cell integrins, *k*, the total number of available receptors, *n*_*r*_, and the concentration of the ligands at the leading edge of the cell, *ψ*. Therefore, it can be defined as [66–68]

$$\begin{array}{c} \zeta =kn_{r}\psi \end{array}$$ (3)

*ζ* depends on the cell type and can be different in the anterior and posterior parts of the cell. Its definition is given in the following sections. Thereby, the net traction force affecting on the whole cell because of cell-substrate interaction can be calculated by [69]

$$\begin{array}{c} F_{\text{net}}^{\text{trac}}=-\sum_{i=1}^{n}F_{i}^{\text{trac}} \end{array}$$ (4)

where *n* is the number of the cell membrane nodes. During migration, nodal traction forces (contraction forces) exerted on cell membrane towards its centroid compressing the cell. Consequently, each finite element node on the cell membrane, which has less internal deformation, will have a higher traction force [69]. On the contrary, the drag force opposes the cell motion through the substrate that depends on the relative velocity and the linear viscoelastic character of the cell substrate. At micro-scale the viscous resistance dominates the inertial resistance of a viscose fluid [75]. Assuming ECM as a viscoelastic medium and considering negligible convection, Stokes’ drag force around a sphere can be described as [76]

$$\begin{array}{c} F_{\text{D}}^{\text{s}}=6\;\pi r\eta \left(E_{\text{sub}}\right)v \end{array}$$ (5)

where *v* is the relative velocity and *r* is the spherical object radius. *η*(*E*_sub_) is the effective medium viscosity. Within a substrate with a linear stiffness gradient, we assume that effective viscosity is linearly proportional to the medium stiffness, *E*_sub_, at each point. Therefore it can be calculated as

$$\begin{array}{c} \eta \left(E_{\text{sub}}\right)=\eta_{\text{min}}+\lambda E_{\text{sub}} \end{array}$$ (6)

where *λ* is the proportionality coefficient and *η*_min_ is the viscosity of the medium corresponding to minimum stiffness. Although, the viscosity coefficient may be finally saturated with higher substrate stiffness, this saturation occurs outside the substrate stiffness range that is proper for some cells [58].

Equation 5 was developed by Stoke to calculate the drag force around a spherical shape object with radius *r*. This typical equation was employed in our previous works for cell migration with constant spherical shape [66, 69]. In the present work, according to Equations 17–19, an inaccurate calculation of the drag force may affect considerably the calculation accuracy of the cell velocity and polarization direction. So that, according to [77, 78], a shape factor is appreciated to moderate the Stokes’ drag expression to be suitable for irregular cell shape. The drag of irregular solid objects depends on the degree of non-sphericity and their relative orientation to the flow. Therefore for an irregular object shape the drag is basically anisotropic compared to movement direction. Since here the objective is to investigate cell migration while cell morphology changes, calculation of the drag force using Equation 5 will not be precise enough. Due to the randomness of the cell shapes and dynamics, description of drag force for objects with irregular shape is extremely complicated. It is thought that only probabilistic and approximate predictions can be reasonable and useful to describe drag force for highly irregular particles [77, 78]. Therefore, referring to experimental observations, an appropriate shape factor, *f*_shape_, is appreciated to moderate the Stokes’ drag expression for highly irregularly-shaped objects which is accurate enough [68, 77, 78]

$$\begin{array}{c} F_{\text{drag}}=f_{\text{shape}}F_{\text{D}}^{\text{s}} \end{array}$$ (7)

A wide variety of shape-characterizing parameters has been suggested for irregular particles. Here we have employed Corey Shape Factor (CSF) which is the most common and accurate shape factor. It appreciates three main lengths of an object that are mutually perpendicularly to each other as

$$\begin{array}{c} f_{\text{shape}}={\left(\frac{l_{\text{max}}l_{\text{med}}}{l_{\text{min}}^{2}}\right)}^{0.09} \end{array}$$ (8)

where *l*_max_, *l*_med_ and *l*_min_ are the cell’s longest, intermediate and the shortest dimensions, respectively, which are representative of cell surface area changes [77]. In the case of a spherical cell shape, this shape factor delivers 1. Although other shape factors have been proposed to characterize the shape irregularity, using the max-med-min length factor leads to reliable results [77, 79].

### Protrusion force

To migrate, cells extend local protrusions to probe their environment. This is the duty of protrusion force generated by actin polymerization which has a stochastic nature during cell migration [80]. It should be distinguished from the cytoskeletal contractile force [68, 75]. The order of the protrusion force magnitude is the same as that of the traction force but with lower amplitude [69, 75, 81–83]. Therefore, we randomly estimate it as

$$\begin{array}{c} F_{\text{prot}}=\kappa F_{\text{net}}^{\text{trac}}e_{\text{rand}} \end{array}$$ (9)

where **e**_rand_ is a random unit vector and $F_{\text{net}}^{\text{trac}}$ is the magnitude of the net traction force while *κ* is a random number, such that 0 ≤ *κ* < 1, [66, 68].

### Electrical force in presence of electrotactic cue

**Fig 1:**
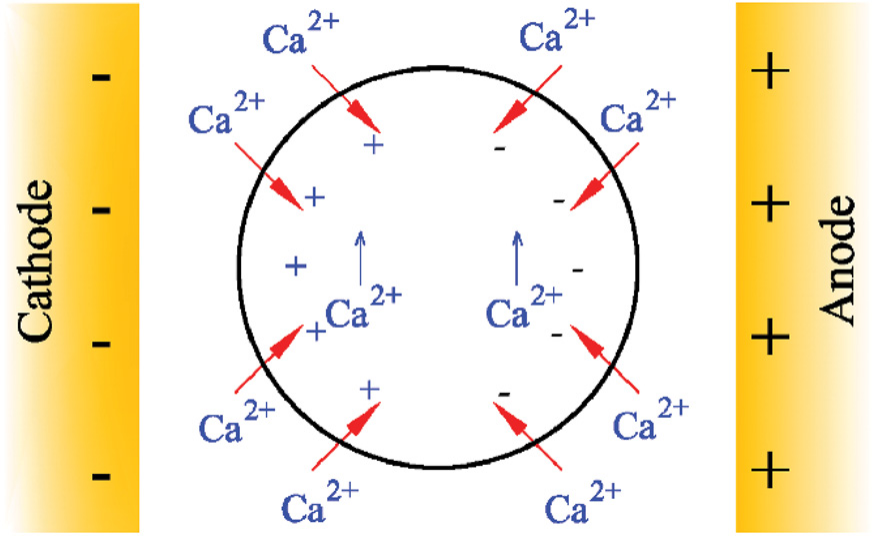
Response of a cell to a dcEFs. A simple cell in the resting state has a negative membrane potential [25]. When a cell with a negligible voltage-gated conductance is exposed to a dcEF, it is hyperpolarised membrane near the anode attracts Ca^2+^ due to passive electrochemical diffusion. Consequently, this side of the cell contracts, propelling the cell towards the cathode. Therefore, voltage-gated Ca^2+^ channels (VGCCs) near cathode (depolarised side) open and a Ca^2+^ influx occurs. In such a cell, intracellular Ca^2+^ level rises in both sides. The direction of cell movement, then, depends on the difference of the opposing magnetic contractile forces, which are exerted by cathode and anode [25].

Exogenous EFs imposed to a cell have been proposed as a directional cue that directs the cells to migrate in cell therapy. Besides, studies in the last decade have provided convincing evidence that there is a role for EFs in wound healing [6]. Significantly, this role is highlighted more than expected due to overriding other cues in guiding cell migration during wound healing [6, 31]. Experimental works demonstrate that Ca^2+^ influx into cell plays a significant role in the electrotactic cell response [25, 26, 28]. Although this is still a controversial open question, Ca^2+^ dependence of electrotaxis has been observed in many cells such as neural crest cells, embryo mouse fibroblasts, fish and human keratocytes [23, 25, 27, 30, 40]. On the other hand, Ca^2+^ independent electrotaxis has been observed in mouse fibroblasts [32]. The precise mechanism behind intracellular Ca^2+^ influx during electrotaxis is not well-known. A simple cell at resting state maintain a negative membrane potential [25] so that exposing it to a dcEF causes that the side of the plasma membrane near the cathode depolarizes while the the other side hyperpolarizes [23, 25, 30]. For a cell with trivial voltage-gated conductance, the membrane side which is hyperpolarized attracts Ca^2+^ due to passive electrochemical diffusion. Therefore, this side of the cell contracts and propels the cell towards the cathode which causes to open the voltage-gated Ca^2+^ channels (VGCCs) near the cathode (depolarised) and allows intracellular Ca^2+^ influx (Fig 1). So, on both anodal and cathodal sides of the cell, intracellular Ca^2+^ level enhances. Balance between the opposing magnetic forces defines the resultant electrical force affecting the cell body [25]. That is the reason that some cells tend to reorient towards the anode, like metastatic human breast cancer cells [84], human granulocytes [85], while some others do towards the cathode, such as human keratinocytes [26, 86], embryo fibroblasts [27], human retinal pigment epithelial cells [87] and fish epidermal cells [40].

A single cell embedded within a uniform EF will be ionized and charged. Therefore the electrical force experienced by this individual cell can be obtained by

$$\begin{array}{c} F_{\text{EF}}=E\;\Omega \left(E\right)\;S\left(t\right)e_{\text{EF}} \end{array}$$ (10)

where *E* is uniform dcEF strength and Ω(*E*) stands for the surface charge density of the cell. **e**_EF_ is a unit vector in the direction of the dcEF toward the cathode or anode, depending on the cell type. The time course of the translocation response during exposing a cell to a dcEF demonstrates that the cell velocity versus translocation varies depending on the dcEF strength. Experiments of Nishimura et al. [26] on human keratinocytes indicate that the net migration velocity raises by increase the dcEF strength to about 100 mV/mm while further increase the dcEF strength does not affect the cell net migration velocity. Since the Ca^2+^ influx into intracellular may play a role in this process [25, 26, 28, 88–90], it is thought that the imposed dcEF regulates the concentration of intracellular Ca^2+^. Therefore, it can be deduced that the cell surface charge is directly proportional to the imposed dcEF strength [25, 26]. Consequently, we assume a linear relationship between the cell surface charge and the applied dcEF strength as

$$\begin{array}{c} \Omega (E)=\left\{\begin{array}{cc} \frac{\Omega_{\text{satur}}}{E_{\text{satur}}}E\; & E\leq E_{\text{satur}}\\ \Omega_{\text{satur}}\; & E>E_{\text{satur}} \end{array}\right. \end{array}$$ (11)

where Ω_satur_ is the saturation value of the surface charge and *E*_satur_ is the maximum dcEF strength that causes Ca^2+^ influx into intracellular.

### Deformation and reorientation of the cell

**Fig 2:**
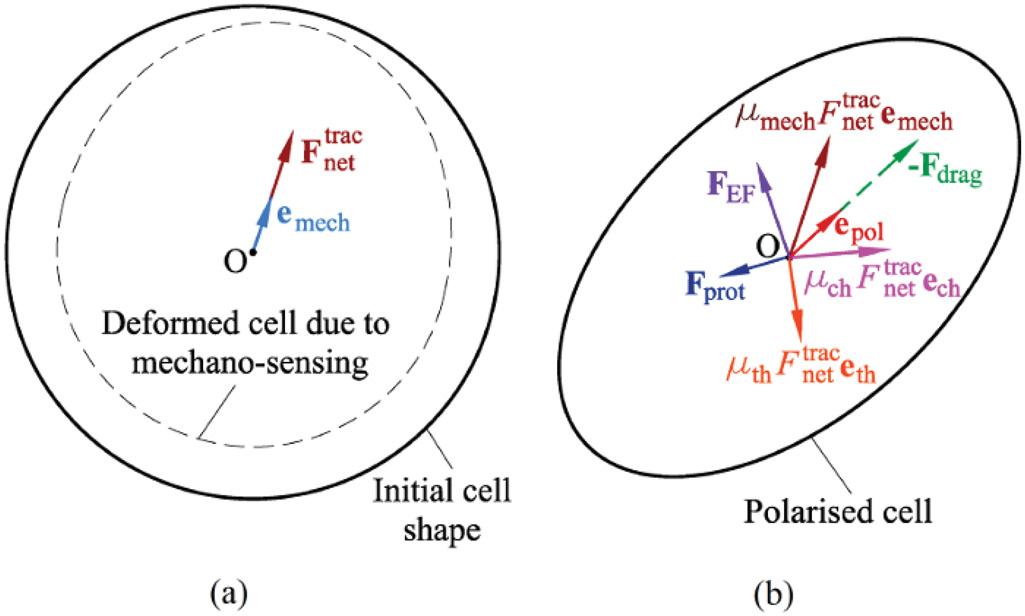
Calculation of the cell reorientation. a- A initially spherical cell (solid line) is deformed (dashed line) during mechano-sensing process. **e**_mech_ is mechanotaxis reorientation of the cell. b- A cell is reoriented due to exposing to chemotaxis, thermotaxis and electrotaxis where **e**_ch_, **e**_th_ and **e**_EF_ denote the unit vector in the direction of each cue, respectively. The coefficients *μ*_mech_, *μ*_ch_, and *μ*_th_ are effective factors of mechanotactic, chemotactic and thermotactic cues, respectively. $F_{\text{net}}^{\text{trac}}$ is the magnitude of the net traction force, **F**_prot_ is the random protrusion force, **F**_EF_ represents the electrical force that is exerted by dcEF and **F**_drag_ stands for drag force. **e**_pol_ represents the net polarisation direction of a cell in a multi-signaling environment.

Solid line in Fig 2 shows a spherical cell configuration which is initially considered. It is assumed that the cell first exerts mechano-sensing forces on the membrane to probe its surrounding micro-environment which is named mechano-sensing process. Thus, the cell internal strain at each finite element node of the cell membrane along **e**_*i*_ can be calculated by

$$\begin{array}{c} \epsilon_{\text{cell}}=e_{i}:\epsilon_{i}:{e_{i}}^{\text{T}} \end{array}$$ (12)

where ***ϵ***_*i*_ is the strain tensor of *i*th node located on cell membrane due to mechano-sensing process.

A cell exerts contraction forces towards its centroid compressing itself so that the cell internal deformation, *ϵ*_cell_, created by these forces on each finite element node of the cell membrane is negative. Hence, according to Equations 1 and 2 nodes with a less internal deformation experience a higher internal stress and traction force. Therefore, the net traction forces, ${\text{F}}_{\text{net}}^{\text{trac}}$, points towards the direction of minimum cell internal deformation (Equation 4), presenting the mechanotaxis reorientation of the cell [69]. Consequently, the unit vector of the mechanotactic reorientation of the cell, **e**_mech_, reads

$$\begin{array}{c} e_{\text{mech}}=\frac{F_{\text{net}}^{\text{trac}}}{\left\|F_{\text{net}}^{\text{trac}}\right\|} \end{array}$$ (13)

In presence of thermotaxis or chemotaxis, the cell polarisation direction will be controlled by all the existent stimuli. It is assumed that the presence of both additional cues does not affect either the physical or the mechanical properties of a typical cell, nor its surrounding ECM. Traction forces exerted by a typical cell depend on the mechanical apparatus of the cell and the mechanical properties of the substrate [21]. Therefore, the mechanotactic tool practically drives the cell body forward while the presence of chemotaxis and/or thermotaxis cues only changes the cell polarisation direction such that a part of the net traction force is guided by mechanotaxis and the rest is guided by these stimuli (Fig 2). Consequently, under chemical and/or thermal gradients, the unit vectors associated to the chemotactic and thermotactic stimuli can be represented, respectively, as [66, 67]

$$\begin{array}{c} e_{\text{ch}}=\frac{\nabla C}{\left\|\nabla C\right\|} \end{array}$$ (14)

$$\begin{array}{c} e_{\text{th}}=\frac{\nabla T}{\left\|\nabla T\right\|} \end{array}$$ (15)

where ∇ denotes the gradient operator while *C* and *T* represent the chemoattractant concentration and the temperature, respectively. As mentioned above, the realignment of the net traction force under these cues is affected by the direction of chemical and thermal gradients, so that the effective force, **F**_eff_, which incorporates mechanotactic, chemotactic and thermotactic effects can be defined as

$$\begin{array}{c} F_{\text{eff}}=F_{\text{net}}^{\text{trac}}\left(\mu_{\text{mech}}e_{\text{mech}}+\mu_{\text{ch}}e_{\text{ch}}+\mu_{\text{th}}e_{\text{th}}\right) \end{array}$$ (16)

where *μ*_mech_, *μ*_ch_ and *μ*_th_ are the effective factors of mechanotaxis, chemotaxis, and thermotaxis cues respectively, *μ*_mech_ + *μ*_ch_ + *μ*_th_ = 1. It is assumed that there is neither degradation nor remodeling of the ECM during cell motility. Having in account that the inertial force is negligible, the cell motion equation delivers drag force as

$$\begin{array}{c} F_{\text{drag}}+F_{\text{eff}}+F_{\text{prot}}+F_{\text{EF}}=0 \end{array}$$ (17)

Thereby, using Equation 7, the instantaneous velocity of the cell is defined as

$$\begin{array}{c} v=\frac{\left\|F_{\text{drag}}\right\|}{f_{\text{shape}}\;6\;\pi r\eta \left(E_{\text{sub}}\right)} \end{array}$$ (18)

with the net polarisation direction

$$\begin{array}{c} e_{\text{pol}}=-\frac{F_{\text{drag}}}{\left\|F_{\text{drag}}\right\|} \end{array}$$ (19)

### Cell morphology and cell remodeling during cell migration

Cell migration composed of several coordinated cyclic cellular processes. At the light microscope level, many authors summarize this process into several steps such as leading-edge protrusion, formation of new adhesions near the front, contraction, releasing old adhesions and rear retraction [11, 91]. At the trailing end the cortical tension squeezes or presses the cytoplasm in the direction of migration while at the leading edge, the tension generated due to protrusions drives the cells forward [3, 92].

**Fig 3:**
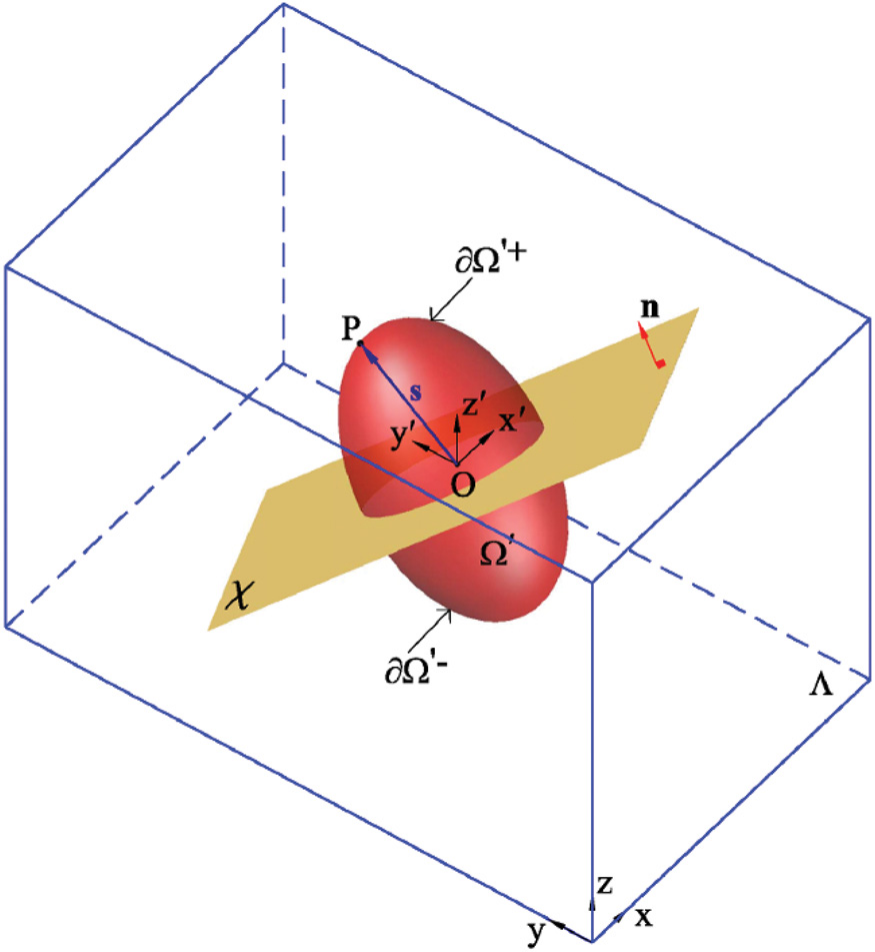
Definition of extension and retraction points as well as anterior and posterior parts of the cell at each time step. Λ ⊂ *R* ^3^, Ω and Ω′ represent the 3D working space, matrix and cell domains, respectively. **X** stands for the global coordinates and **X**′ represents the local cell coordinates located in the cell centroid, O. *χ* is a plane passing by the cell centroid with unit normal vector **n** parallel to the cell polarisation direction, **e**_pol_. P denotes a finite element node located on the cell membrane, ∂Ω. ∂Ω′^+^ and ∂Ω′^−^ are the finite element nodes located on the front and rear of the cell membrane, respectively.

Guided by the aforementioned experimental observations, the regulatory process behind the cell shape during cell migration is here simplified to analyze cell shape changes coupled with the cell traction forces. Therefore, we model the dominant modes of cell morphological changes considering the cell body retraction at the rear and extension at the front. Referring to Fig 3, the initial domain of the cell, which is located within the working space of Λ ⊂ *R* ^3^ with the global coordinates of **X**, may be described as

$$\begin{array}{c} \Omega \prime =\{x\prime \left(\text{X}\prime \right)|x\prime \left(\text{X}\prime \right)\in \Lambda :\forall \|x\prime \left\|⩽r\right\} \end{array}$$ (20)

where **X**′ denotes the local cell coordinates located in the cell centroid. Accordingly the cell membrane can be represented by ∂Ω′. Thereby, the substrate domain can be defined as

$$\begin{array}{c} \Omega =\{x(\text{X})|x(\text{X})\in \Lambda ,\;x(\text{X})\notin \Omega \prime \} \end{array}$$ (21)

During cell migration, both domains Ω′ and Ω vary such that Ω′ ∪ Ω = Λ and Ω′ ∩ Ω = ∅.

To correctly incorporate adhesivity, *ζ*, of cell in the cell front and rear, it is essential to define the cell anterior and posterior during cell motility. Assuming *χ* is a plane passing by the cell centroid, O, with unit normal vector **n**, parallel to **e**_pol_, and **s**(**X**′) is a position vector of an arbitrary node located on ∂Ω′ (Fig 3), projection of **s** on **n** can be defined as

$$\begin{array}{c} \delta =n\cdot s \end{array}$$ (22)

Consequently, nodes with positive *δ* are located on the cell membrane at the front, ∂Ω′^+^, while nodes with negative *δ* belong to the cell membrane at the cell rear, ∂Ω′^−^, where ∂Ω′ = ∂Ω′^+^ ∪ ∂Ω′^−^ should be satisfied.

We assume that the cell extends the protrusion from the membrane vertex whose position vector is approximately in the direction of cell polarisation, on the contrary, it retracts the trailing end from the membrane vertex whose position vector is totally in the opposite direction of cell polarisation. Thus, the maximum value of *δ* delivers the membrane node located on ∂Ω′^+^ from which the cell must be extended while the minimum value of *δ* represents the membrane node located on ∂Ω′^−^ from which the cell must be retracted. Assume *e* ^ex^ ∈ Ω is the finite element that the membrane node with the maximum value of *δ* belongs to its space and *e* ^re^ ∈ Ω′ is the finite element that the membrane node with the minimum value of *δ* belongs to its space. To integrate cell shape changes and cell migration, simply, *e* ^re^ is moved from the Ω′ domain to the Ω domain, in contrast, *e* ^ex^ is eliminated from the Ω domain and is included in the Ω′ domain [68].

In the present model the cell is not allowed to obtain infinitely thin shape during migration. Therefore, consistent with the experimental observation of Wessels et al. [93, 94], it is considered that the cell can extend approximately 10% of its whole volume as pseudopodia.

## Finite element implementation

**Fig 4:**
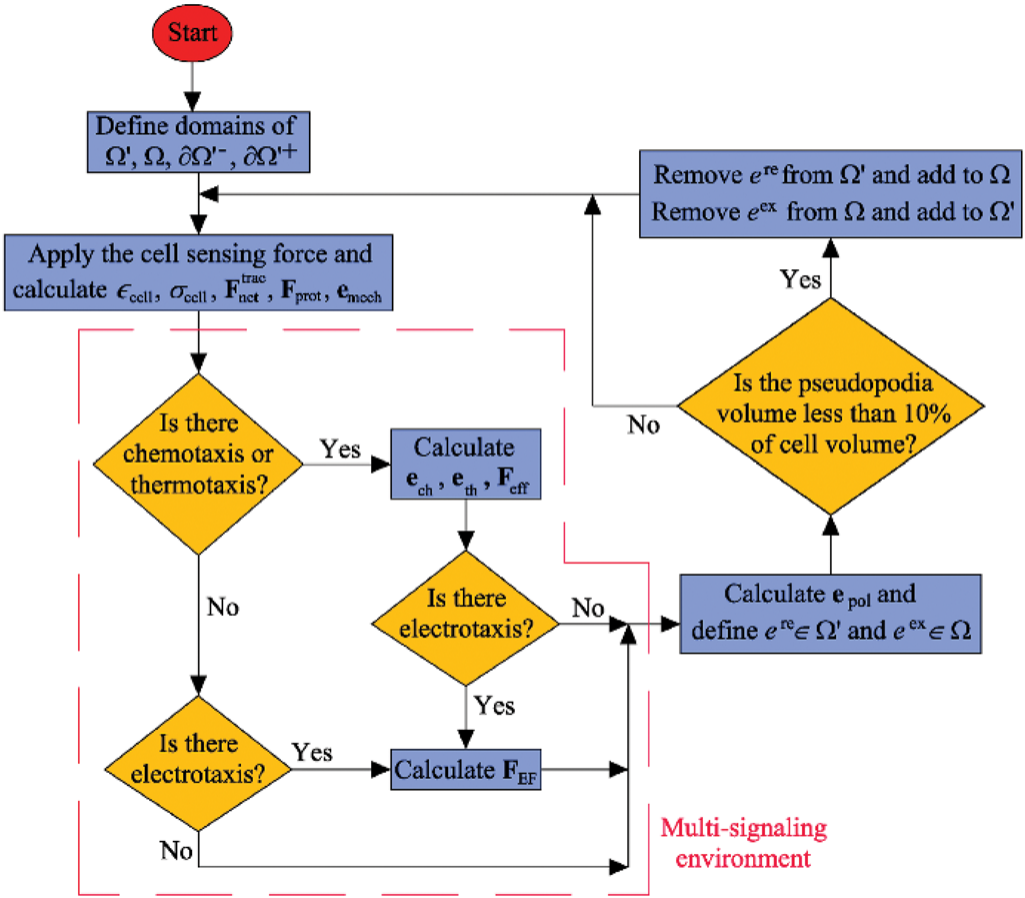
Computational algorithm of migration and cell morphology changes in a multi-signaling environment.

The present model is implemented through the commercial finite element (FE) software ABAQUS [95] using a coupled user element subroutine. The corresponding algorithm is presented in Fig 4.

The model is applied in several numerical examples to investigate cell behavior in the presence of different stimuli. It is assumed that the cell is located within a 400×200×200 *μ*m matrix without any external forces. The matrix is meshed by 128,000 regular hexahedral elements and 136,161 nodes while the cell is represented by 643 elements. The calculation time is about one minute for each time step in which each step corresponds to approximately 10 minutes of real cell-matrix interaction [68]. Initially the cell is assumed to have a spherical shape as shown in Fig 2a. In Table 1, the properties of the matrix and the cell are enumerated.

**Table 1:** 3D matrix and cell properties.

**Symbol**	**Description**	**Value**	**Ref.**
*ν*	Poisson ratio	0.3	[97, 98]
*μ*	Viscosity	1000 Pa⋅s	[75, 97]
*r*	Cell radius	20 *μ*m	[99]
*K*_*pas*_	Stiffness of microtubules	2.8 kPa	[100]
*K*_*act*_	Stiffness of myosin II	2 kPa	[100]
*ϵ*_*max*_	Maximum strain of the cell	0.09	[69, 83]
*ϵ*_*min*_	Minimum strain of the cell	-0.09	[69, 83]
*σ*_*max*_	Maximum contractile stress exerted by actin-myosin machinery	0.1 kPa	[101, 102]
*k*_*f*_ = *k*_*b*_	Binding constant at the rear and at the front of the cell	10^8^ mol^−1^	[75]
*n*_*f*_ = *n*_*b*_	Number of available receptors at the rear and at the front of the cell	10^5^	[75]
*ψ*	Concentration of the ligands at the rear and at the front of the cell	10^−5^ mol	[75]
Ω	Order of surface charge density of the cell	10^−4^ C/m^2^	[24]
*E*	Range of applied electric field	0–100 mV/mm	[25, 30]

For each simulation it is of interest to quantify the cell shape. Therefore, two parameters are calculated to quantify the cell shape changes during cell migration in 3D multi-signaling matrix: Cell Morphological Index (CMI)

$$\begin{array}{c} \text{CMI}\left(t\right)=\frac{S(t)}{S_{\text{in}}} \end{array}$$ (23)

where *S*_in_ denotes the initial area of cell membrane (spherical cell shape); and the cell elongation

$$\begin{array}{c} \epsilon_{\text{elong}}=1-\frac{\sqrt{l_{\text{min}}l_{\text{med}}}}{l_{\text{max}}} \end{array}$$ (24)

Here the second term of the equation represents the ratio of the geometric mean over the cell length. *ϵ*_elong_ is a representative value of cell elongation. It is calculated to evaluate a spherical cell shape versus an elongated cell configuration according to the experimental work of Lee et al [96]. According to Equation 24, *ϵ*_elong_ = 0 for a spherical cell configuration, in contrast for a highly elongated cell, *ϵ*_elong_ ≃ 1. This means that the cell length in one direction is much higher than that of other two mutual perpendicular directions. On the other hand, CMI is another parameter to show how the cell surface area changes during cell migration. In our cases study, we assume that the cell initially has a spherical shape (CMI = 1). This value goes to increase while cell migrates. Therefore, although there is no direct relation between *ϵ*_elong_ and CMI, they may follow the same trend during cell migration. So, both parameters are minimum for a spherical cell shape and maximum for an elongated cell shape. These variables are probed versus cell position (the cell centroid translocation) in each step to see how the cell elongation and surface area change during cell migration in presence of different stimuli.

In addition, the cellular random alignment in a 3D matrix with a cue gradient (stiffness, thermal and/or chemical gradients) or dcEF can be assessed by the angle between the net polarisation direction of the cell and the imposed gradient direction or EF direction, *θ*. Therefore, the Random Index (RI) can be described by

$$\begin{array}{c} \text{RI}=\frac{\sum_{i=1}^{N}\text{cos}\theta_{i}}{N} \end{array}$$ (25)

where *N* represents the number of time steps during which the cell elongation does not change considerably (the cell reaches steady state). RI = -1 indicates totally random alignment of the cell while RI = +1 represents perfect alignment of the cell in direction of the cue gradient or EF direction. Consequently, in the presence of a cue gradient or dcEF, the closer RI to +1, the lower the cell random orientation.

## Numerical examples and results

During cell migration, amoeboid mode of cells causes frequent changes in cell shape as a result of the extension and retraction of protrusions [20]. To consider this, four different categories of numerical examples have been represented to consider cell behavior in presence of different stimuli. All the stimuli such as thermotaxis, chemotaxis and electrotaxis are considered within the matrix with a linear stiffness gradient and free boundary surfaces. It is assumed that, initially, the cell has a spherical configuration. Each simulation has been repeated at least 10 times to evaluate the results consistency.

### Cell behavior in a 3D matrix with a pure mechanotaxis

Experimental investigations demonstrate that cells located within 3D matrix actively migrate in direction of stiffness gradient towards stiffer regions [103]. In addition, it has been observed that during cell migration towards stiffer regions, the cell elongates and subsequently the cell membrane area increases [13, 96].

**Fig 5:**
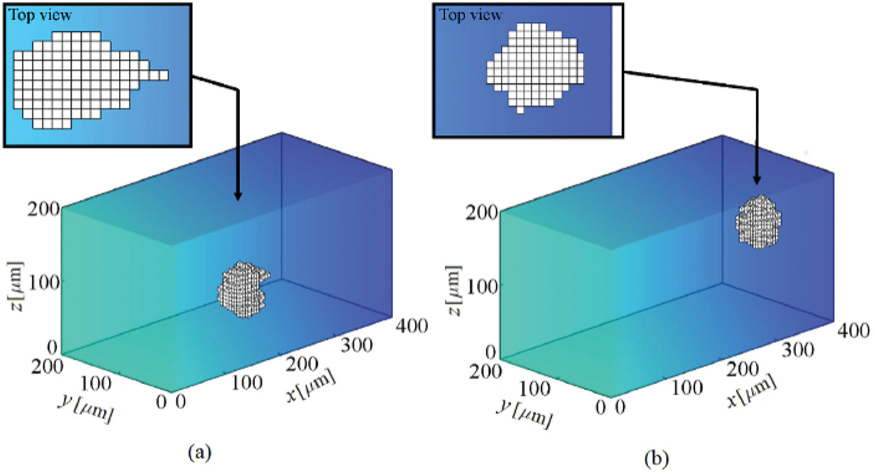
Shape changes during cell migration within a substrate with a linear stiffness gradient. The substrate stiffness changes linearly in *x* direction from 1 kPa at *x* = 0 to 100 kPa at *x* = 400 *μ*m. At the beginning the cell is located at the corner of the substrate near the soft region. The results demonstrate that the cell migrates in the direction of stiffness gradient and the cell centroid finally moves around an IEP located at *x* = 351 ± 5 *μ*m. a- The cell at the middle of the substrate, b- the cell final position (see also S1 Video).

**Fig 6:**
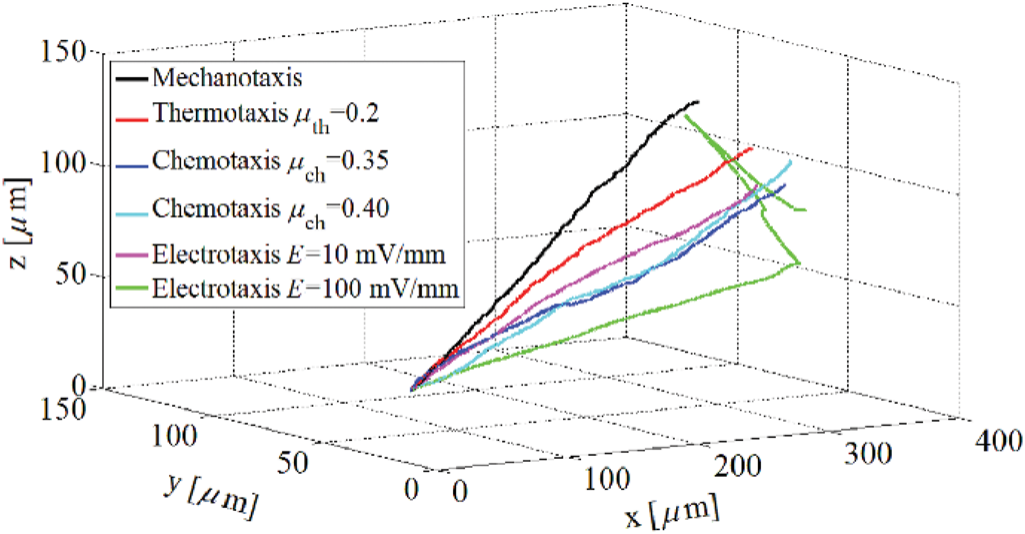
Trajectory of the cell centroid within a substrate with stiffness gradient in presence of different stimuli. Examples are run 10 times in order to check consistency of the results. The slop of the cell centroid trajectory reflects the attractivity of every cue to the cell.

**Fig 7:**
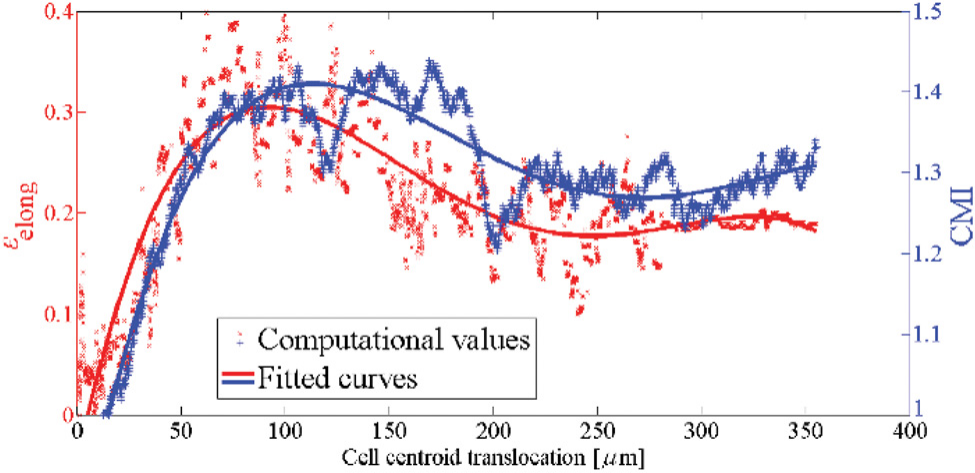
Cell elongation, *ϵ*_elong_ (left axis), and CMI (right axis) versus the cell centroid translocation within a substrate with a pure stiffness gradient. As the cell approaches the intermediate regions of the substrate (rigid regions) both the *ϵ*_elong_ and CMI increase. On the contrary, they decrease near the surface with maximum stiffness because the cell retracts protrusions due to unconstrained boundary surface.

**Fig 8:**
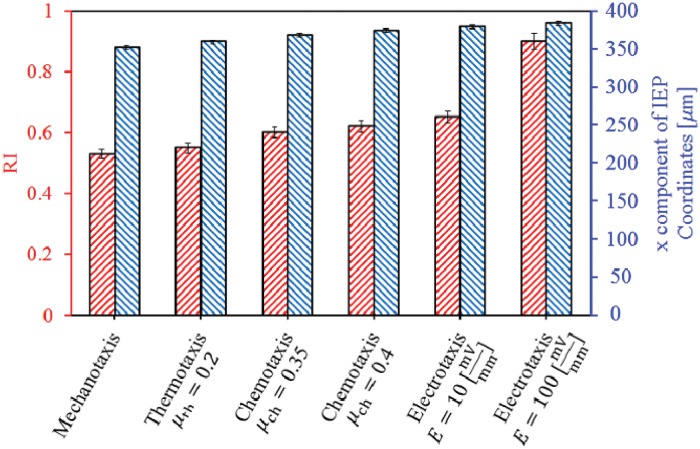
Mean RI (left axis) and IEP position (right axis) of cell in the presence of different cues. The error bars represent mean standard deviation among different runs. Adding a new stimulus to the substrate with stiffness gradient decreases the cell random alignment (increases mean RI) and moves the cell towards the end of the substrate.

To consider the effect of mechanotaxis on cell behavior, it is assumed that there is a linear stiffness gradient in *x* direction which changes from 1 kPa at *x* = 0 to 100 kPa at *x* = 400 *μ*m. The cell is initially located at a corner of the matrix near the boundary surface with lowest stiffness. Fig 5 and Fig 6 show the cell configuration and the trajectory tracked by the cell centroid within a matrix with stiffness gradient, respectively. As expected, independent from the initial position of the cell, when the cell is placed within a substrate with pure stiffness gradient it tends to migrate in direction of the stiffness gradient towards the stiffer region and it becomes gradually elongated. The cell experiences a maximum elongation in the intermediate region of the substrate since it is far from unconstrained boundary surface which is discussed in the previously presented work [66]. As the cell approaches the end of the substrate the cell elongation and CMI decrease (see Fig 7). Despite the boundary surface at *x* = 400 *μ*m has maximum elastic modulus, due to unconstrained boundary, the cell does not tend to move towards it and maintains at a certain distance from it. The cell may extend random protrusions to the end of the substrate but it retracts again and maintains its centroid around an imaginary equilibrium plane (IEP) located far from the end of the substrate at *x* = 351 ± 5 *μ*m (see Fig 8) [69]. Therefore, the cell never spread on the surface with the maximum stiffness. It is worth noting that the deviation of the obtained IEP coordinates is due to the stochastic nature of cell migration (random protrusion force). Fig 8 represents cell RI for the imposed stiffness gradient slope. The simulation was repeated for several initial positions of the cell and several values of the gradient slope, all the obtained results were consistent. However, change in the gradient slope can change the cell random movement and slightly displace the IEP position (results of different gradient slopes are not shown here). Cell behavior within the substrate with stiffness gradient is in agreement with experimental observations [13, 96, 103] and the results of the previous works presented by the same authors in which a constant spherical configuration has been considered for the cell [67, 69]. It is worth mentioning that the net cell traction force and velocity curves are not presented here since they roughly follow the same trend as the previous work [67].

### Cell behavior in presence of thermotaxis

**Fig 9:**
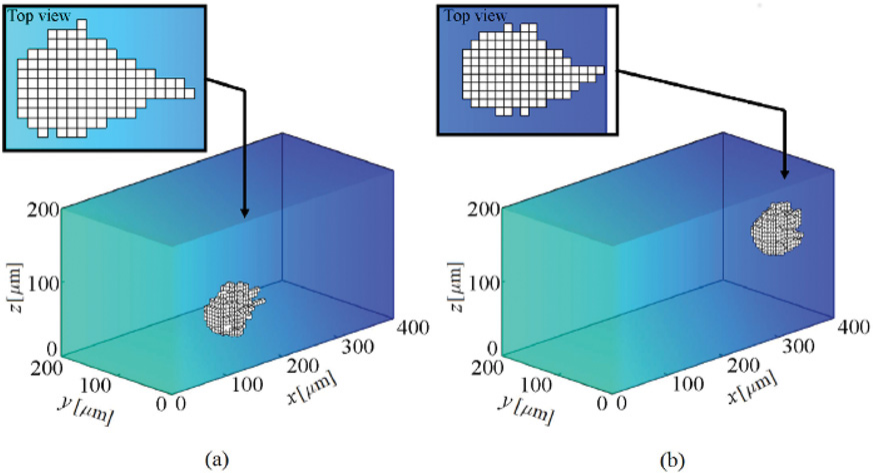
Shape changes during cell migration within a substrate with conjugate linear stiffness and thermal gradients. It is assumed that there is a linear thermal gradient in *x* direction (as stiffness gradient) which changes from 36°C at *x* = 0 to 39°C at *x* = 400 *μ*m. At the beginning the cell is located at a corner of the substrate near the surface with lower temperature. The results demonstrate that the cell migrates along the thermal gradient towards warmer region. Finally, the cell centroid moves around an IEP located at *x* = 359 ± 3 *μ*m. When the cell centroid is near the IEP the cell may send out and retract protrusions but it maintains the position around IEP. a- The cell at the middle of the substrate, b- the cell final position (see also S2 Video).

**Fig 10:**
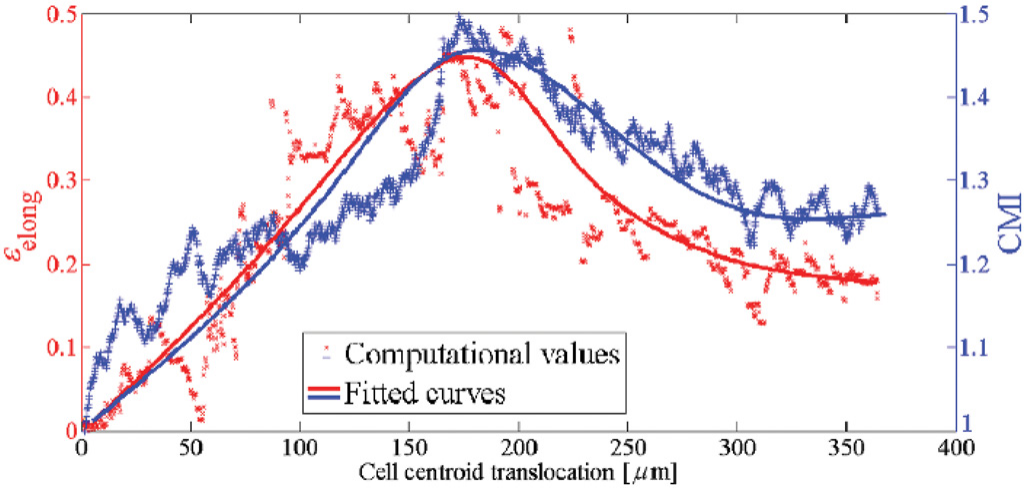
Cell elongation, *ϵ*_elong_ (left axis), and CMI (right axis) versus the cell centroid translocation in the presence of thermotaxis. The cell elongation and CMI are maximum in the intermediate regions of the substrate and decreases as the cell approaches the unconstrained surface with higher temperature.

Several experimental studies [18, 19] have demonstrated that, *in vivo*, different cell types are affected by thermal gradient. Here, employing the present model, we investigate that how the cell can sense and respond to the presence of thermal gradient in its substrate. To do so, a thermal gradient is added to the aforementioned substrate with stiffness gradient. It is assumed that the temperature at *x* = 0 is equal to 36°C and at *x* = 400 *μ*m is 39°C [19], while *μ*_th_ = 0.2. This creates a linear thermal gradient throughout the substrate along *x* axis. At the beginning, the cell is located at one of the corners of the substrate near the boundary surface with minimum temperature. The results indicate that the cell gradually elongates and migrates towards warmer zone in direction of the thermal gradient by means of thermotaxis (Fig 9). Fig 6 demonstrates the trajectory that is tracked by the cell centroid. In this case also there is an IEP located at *x* = 359 ± 3 *μ*m (Fig 8) that the cell centroid finally move around it. Comparing the trajectory of the cell centroid in the presence of thermotaxis with that of pure mechanotaxis indicates that the cell centroid slightly moves towards the end of the substrate with greater temperature. Once the cell achieves IEP, it extends protrusions randomly in different directions maintaining the position of the cell centroid near the IEP. These findings are independent from the initial cell position and are consistent with experimental findings of Higazi et al. [19] who demonstrated that trophoblasts migrate towards warmer locations due to thermal gradient. Comparing RIs of mechanotaxis and thermotaxis cases in Fig 8 illustrates that adding thermotaxis cue to the substrate with stiffness gradient causes decrease in cell random motility (increase in RI). Because mechanical and thermal gradients, which are in the same directions, contribute with each other to more directionally guide the cell. Both the cell elongation and the CMI follow the same trend as mechanotaxis example but in average there is an increase in their amount, which means the contribution of mechanotaxis and thermotaxis increases the cell elongation and the CMI (Fig 10). The thermal gradient imposed here may be considered as the maximum biological gradient, which is applicable in cell environment. We have repeated the simulation for mild thermal gradients but there is no considerable deviation in results (results not shown). Therefore, it can be deduced that the variation of gradient slope in thermotaxis do not dramatically affect the final results because in biological ranges, sharp thermal gradients are not applicable. However, it is noticeable that the cell does not exhibit significant thermotactic response to a very mild thermal gradients (when difference between maximum and minimum temperatures is less than 0.2°C in the substrate).

### Cell behavior in the presence of chemotaxis

Many experimental investigations have demonstrated that the cell has a directional migratory capability in presence of a shallow chemoattractant gradient within 3D surrounding substrates [16, 104]. *In vitro*, observations indicate that cells include a strong basal pseudopod cycle by which pseudopod extension occurs along chemical gradient at the close side of the cell to the higher chemical concentration [20]. This means that the cell elongates its body in direction of chemical gradient towards the higher concentration of chemoattractant substance.

**Fig 11:**
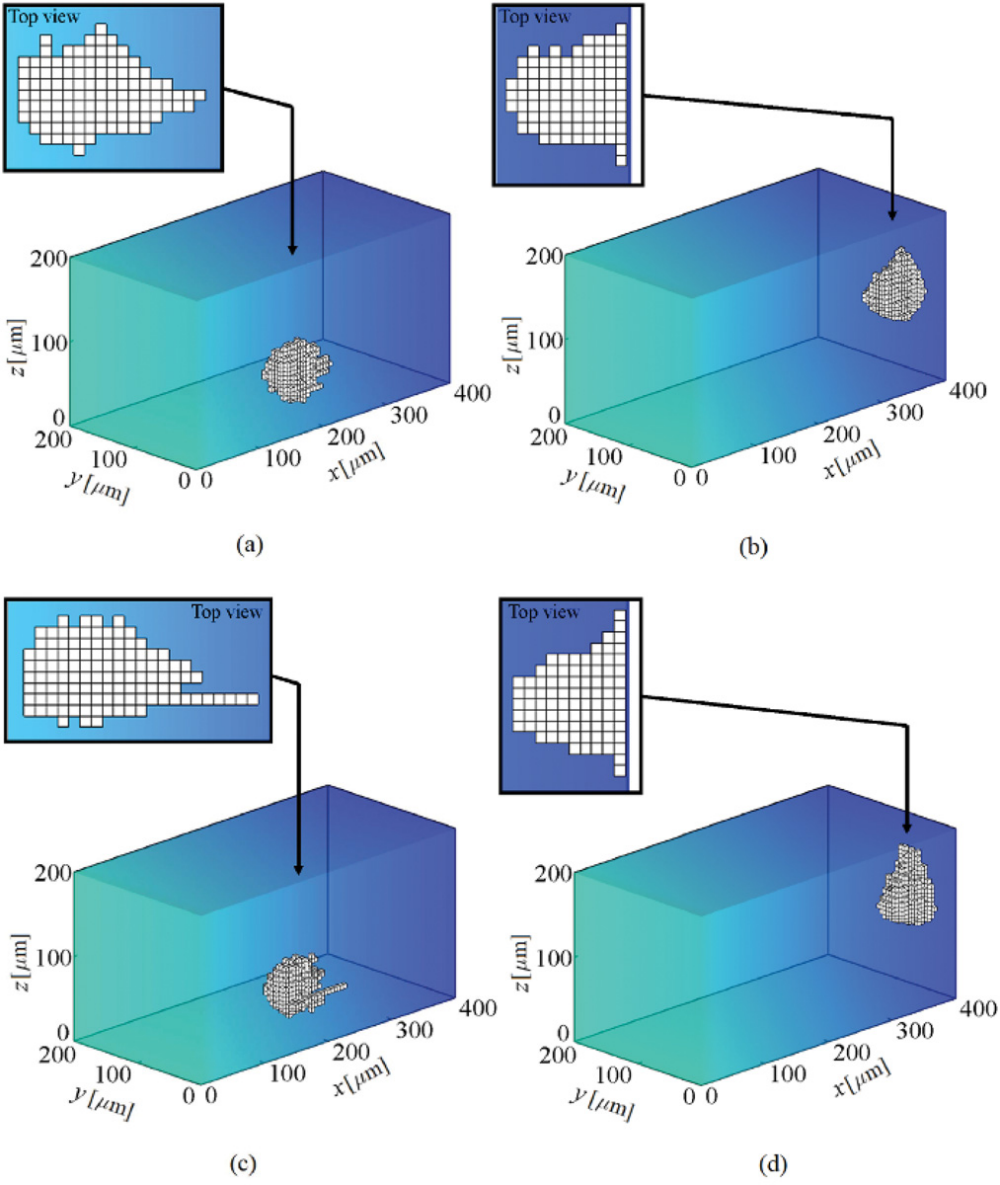
Shape changes during cell migration in presence of chemotaxis within a substrate with stiffness gradient. It is assumed that there is a chemoattractant substance with concentration of 5×10^−5^ M at *x* = 400 *μ*m, which creates a linear chemical gradient across *x* direction. At the beginning the cell is located at one of the corners of the substrate near the surface of null chemoattractant substance. Two chemotaxis effective factors are considered; *μ*_ch_ = 0.35 (a and b) and *μ*_ch_ = 0.4 (c and d). The results demonstrate that, for both cases, the cell migrates along the chemical gradient towards the higher chemoattractant concentration. Depending on chemical effective factor, the ultimate position of the cell centroid will be different, for *μ*_ch_ = 0.35 the cell centroid keeps moving around an IEP located at *x* = 368 ± 3 *μ*m (b) while for higher chemical effective factor, *μ*_ch_ = 0.4, the position of the IEP moves towards chemoattractant source to locate at *x* = 374 ± 4 *μ*m (d). It is remarkable that in both cases the IEP displaces further towards the end of substrate in comparison with thermotaxis case (see also S3 and S4 Videos for low and high chemical effective factors, respectively).

**Fig 12:**
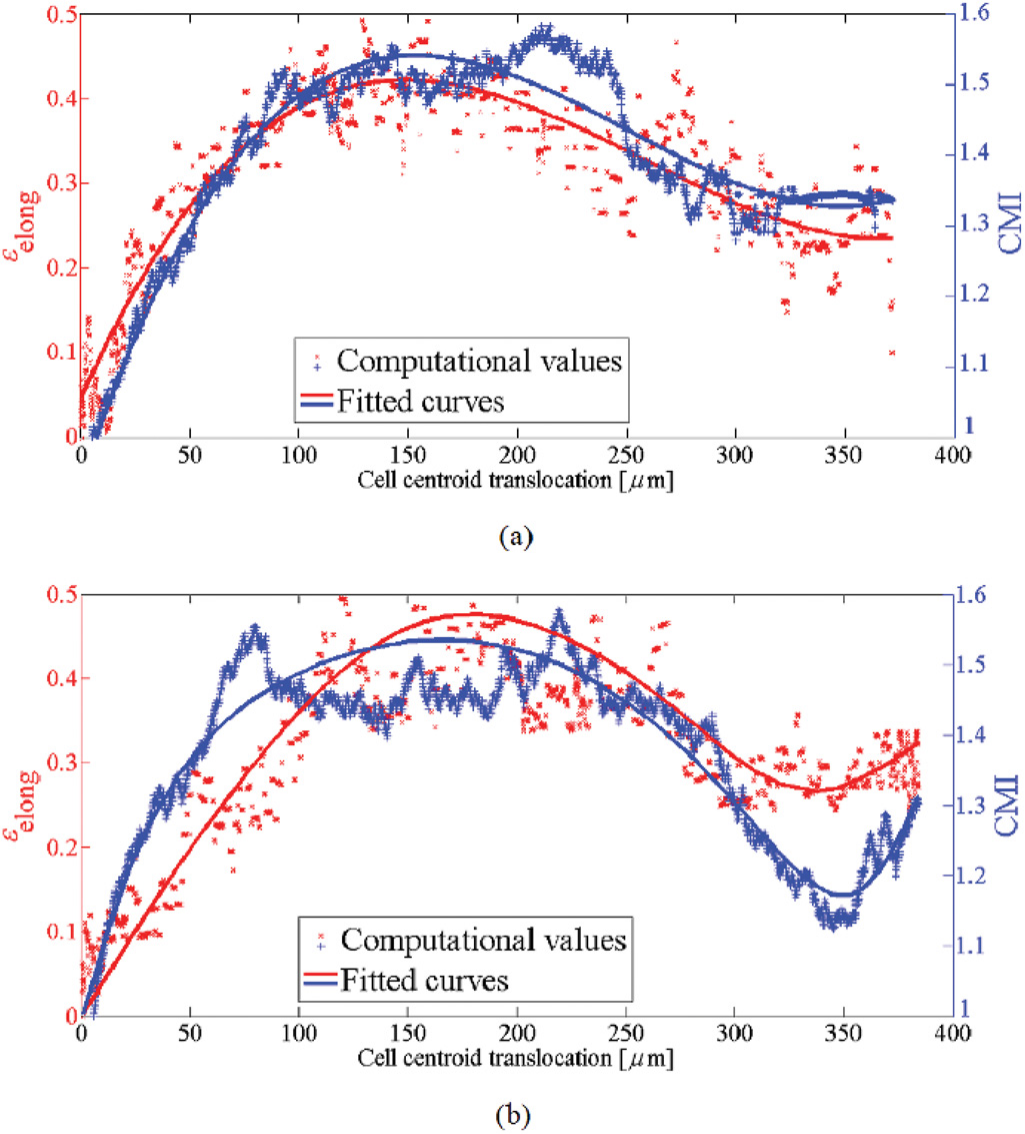
Cell elongation, *ϵ*_elong_ (left axis), and CMI (right axis) versus the cell centroid translocation in the presence of chemotaxis as well as mechanotaxis. a- *μ*_ch_ = 0.35 and b- *μ*_ch_ = 0.40. For both cases, the cell elongation and CMI are maximum in the intermediate regions of the substrate and decreases as the cell approaches the unconstrained surface with chemoattractant source. Because, when the cell reaches the surface with maximum chemoattractant concentration, it tends to adhere to and spread over that surface. However, in the case of chemotaxis cue with higher effective factor the cell again elongates in perpendicular direction to the imposed chemical gradient.

Here, to consider effect of chemotaxis on cell behavior, a chemical gradient is added into the same substrate with stiffness gradient. It is assumed that a chemoattractant substance with concentration of 5×10^−5^ M exists at *x* = 400 *μ*m while chemoattractant concentration at *x* = 0 *μ*m is null. This creates a linear chemical gradient along the *x* axis. The evolution of shape changes during cell migration in the presence of chemotaxis is presented in Fig 11 for two different chemotaxis effective factors, *μ*_ch_ = 0.35 and *μ*_ch_ = 0.4. In Fig 6, the trajectory, which is tracked by the cell centroid, is compared with that of the previous experiments. It implies that the cell centroid ultimately moves around an IEP located at *x* = 368 ± 3 *μ*m and *x* = 374 ± 4 *μ*m for *μ*_ch_ = 0.35 and *μ*_ch_ = 0.4, respectively, (Fig 8). Therefore, it can be deduced that adding a chemotactic stimulus to the substrate moves the final position of the cell centroid towards the chemoattractant source, of course depending on the employed chemotactic effective factor. Similar behavior of cell motility has been observed in the previously presented work by the same authors in which the cell has been represented by a constant spherical shape [67]. In both cases, when the cell is near to the chemoattractant source, it may extend or retract protrusions in random directions, no cell tendency to leave the IEP. It is clear from Fig 12 that for both cases the cell follows the same trend as that of the previous examples in terms of the cell elongation and CMI. However, here, the peak of the cell elongation and CMI slightly increases in comparison with mechanotaxis and/or thermotaxis. In the presence of chemotaxis, the cell tends to spread on the surface on which chemoattractant source is located. It causes cell elongation and CMI increase in perpendicular direction to the imposed chemical gradient, which is considerable in case of greater chemotaxis effective factor (see Figs 11d and 12a). Because of the higher chemotaxis effective factor, the cell receives stronger chemotactic signal to spread more on the surface with chemoattractant source. Besides, the cell random movement relatively decreases for both cases in comparison with either mechanotaxis or thermotaxis example (Fig 8).

Cell migration towards chemoattractant source is qualitatively consistent with many experimental [20, 105, 106] and numerical [17, 51, 107] studies. Besides, cell elongation and shape change during migration is consistent with finding of Maeda et al. [108] implying that gradient sensing and polarization direction of the cell are linked to the cell shape changes and accompanied with motility length of pseudopods.

### Cell behavior in presence of electrotaxis

As mentioned above, endogenous EF is developed around wounds during tissues injury, causing cell migration towards wound cites. Experiments show that in a Guinea pig skin injury just 3 mm away from wound, lateral potential drops to 0 from 140 mV/mm at the wound edge [6, 109–111]. Besides, in cornea ulcer, an EF equal to 42 mV/mm is measured [6, 112]. The cell movement can be also directed and accelerated via exposing it to an exogenous dcEF depending on cell phenotype. In this process, both calcium ion release from and influx into intracellular are generally associated with cell polarisation direction. For instance, human granulocytes [85], rabbit corneal endothelial cells [113], metastatic human breast cancer cells [84] are attracted by anode. Unlike metastatic rat prostate cancer cells [114], embryo fibroblasts [27], human keratinocytes [86], fish epidermal cells [40], human retinal pigment epithelial cells [87], epidermal and human skin cells [30] that move towards cathode. Therefore, altogether, different cell phenotypes may present different electrotactic behavior.

**Fig 13:**
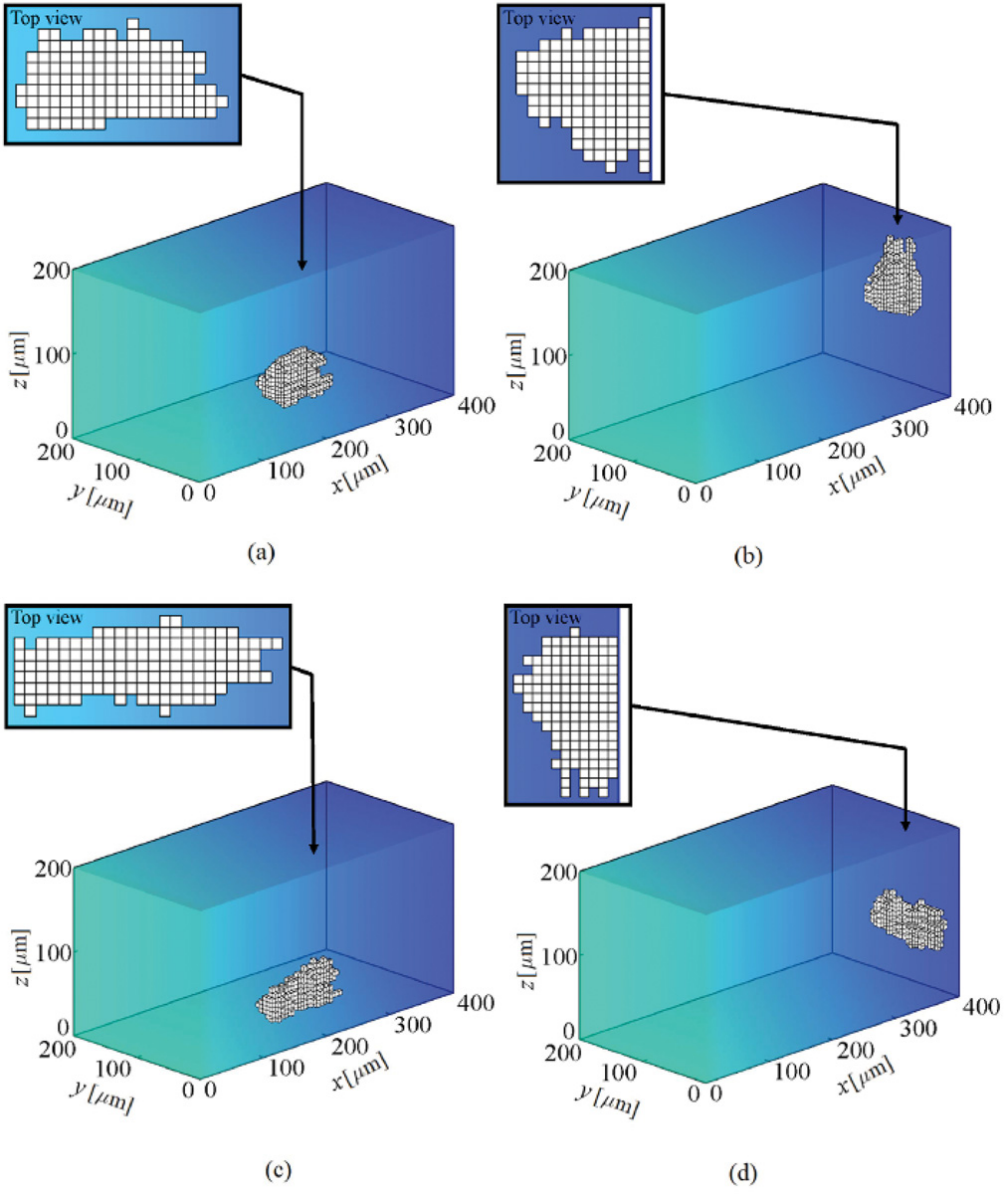
Shape changes during cell migration in presence of electrotaxis within a substrate with stiffness gradient. A cell is exposed to a dcEF where the anode is located at *x* = 0 and the cathode at *x* = 400 *μ*m. It is supposed that the cell is attracted to the cathode pole. At the beginning, the cell is placed in one of the corners of the substrate near the anode and far from the cathode pole. Two EF strength are considered; *E* = 10 mV/mm (a and b) and *E* = 100 mV/mm (c and d). For both cases, the cell migrates along the dcEF towards the surface in which the cathode pole is located. Depending on EF strength, the ultimate location of the cell centroid will be different so that for *E* = 10 mV/mm the cell centroid keeps moving around an IEP located at *x* = 379 ± 3 *μ*m (b) while for saturation EF strength, *E* = 100 mV/mm, the position of the IEP moves further to the cathode pole to locate at *x* = 383 ± 2 *μ*m (d). In the case of saturation EF strength (*E* = 100 mV/mm) the cell perfectly elongates on the surface of cathode pole without extending any protrusion (see also S5 and S6 Videos for low and high EF strengths, respectively).

**Fig 14:**
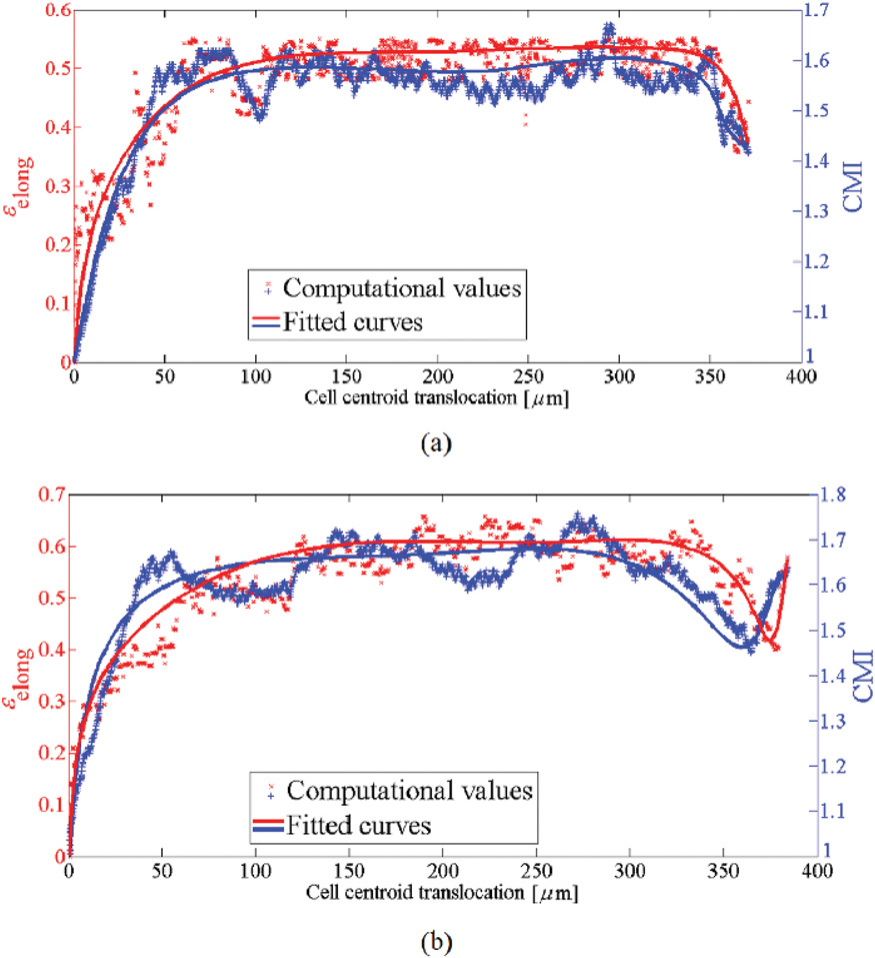
Cell elongation, *ϵ*_elong_ (left axis), and CMI (right axis) versus the cell centroid translocation in the presence of electotaxis as well as mechanotaxis. a- *E* = 10 mV/mm and b- *E* = 100 mV/mm. The cell elongation and CMI reaches a maximum amount sooner than previous cases and are aproximately constant until the cell reaches the cathode pole. The cell elongation and CMI decrease when the cell reaches the surface on which the cathode pole is located but they never diminish less than those of other stimuli. However, in the case of higher EF strength the cell elongation and CMI again increase. The cell elongation and CMI are maximum in this case compared to the other previous cases.

To consider the influence of the electrotaxis on cell behavior, it is considered that the cell is exposed to a dcEF through which the anode is located at *x* = 0 *μ*m and the cathode at *x* = 400 *μ*m. It is assumed that the cell phenotype is such that to be attracted by the cathode, such as human keratinocytes [86] or embryo fibroblasts [27]. First, the cell is located near the anode at *x* = 0. To demonstrate effect of dcEF strength on cell behavior the simulation is repeated for two different dcEF strength, *E* = 10 mV/mm and *E* = 10 100 mV/mm. Cell migration and shape change in the presence of both weak and strong EF are presented in Fig 13. In response of an EF, the cell re-organizes its side that is facing the cathode, and migrates directionally towards the cathode. The presence of the EF can dominate mechanotaxis effect and move the cell to the end of the substrate even more than previous cases where the cell centroid locates around IEP at *x* = 379 ± 3 *μ*m and *x* = 383 ± 2 *μ*m for the weak and strong EF strengths, respectively, (Fig 6 and Fig 8). Besides, the presence of the EF decreases considerably the random movement of the cell (see Fig 8). Near the cathode pole in the presence of weak EF the cell may extend many protrusions in different directions but the change of the cell centroid position is trivial. This is not the case in presence of strong EF, the position of the cell centroid remains constant due to the domination EF role. The cell is even unable to send out any protrusion. This takes place because the strong EF provides a dominant directional signal to guide the migrating cell towards the cathode, dominating the effect of other forces. This is consistent with previous work presented by the same authors assuming constant spherical cell shape [67] where the cell became immobile when it reaches the cathode in the presence of stronger EF strength. EF induces morphological change in the migrating cell where for both cases the average cell elongation and CMI are higher than those of all the previous cases (Fig 14). In presence of electrotaxis the cell achieves the maximum elongation sooner than the other cases and it maintains the maximum amounts until it reaches the end of substrate. Therefore, a flat region can be seen in the fitted elongation and CMI curves (Fig 14). For both cases, near the cathode, the cell elongation and CMI decrease, because in the presence of dcEF the cell tends to spread on the surface where the cathodal pole is located. However, in case of strong EF the cell elongation and CMI again increases because the electrical force acting on the cell body is strong enough to cause the cell elongation perpendicularly to dcEF direction, leading increase in the cell elongation and CMI. It is noteworthy mentioning that for both cases the ultimate cell elongation and CMI are greater that all previous studied cases.

### Cell shape change in Multi-signalling substrate

**Fig 15:**
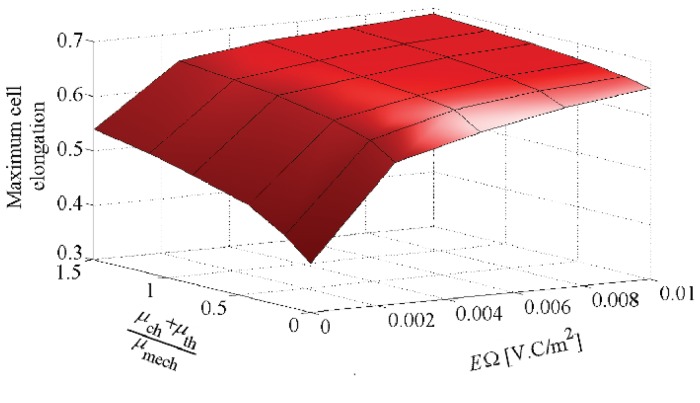
Variation of the maximum cell elongation, *ϵ*_elong_, versus thermotaxis, chemotaxis and electrotaxis stimuli.

**Fig 16:**
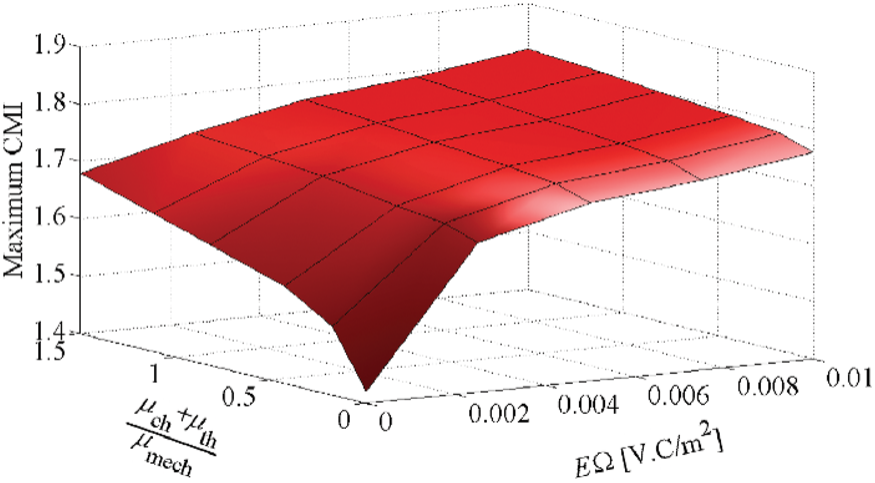
Variation of the maximum CMI versus thermotaxis, chemotaxis and electrotaxis stimuli.

Finally, to simultaneously evaluate the effect of different stimuli on cell shape change during cell migration, we have designed 30 different cases through which different thermotaxis and chemotaxis effective factors as well as different EF strengths are applied. The maximum cell elongation, *ϵ*_elong_, and CMI versus the combination of stimuli, which occur in the intermediate area of the substrate, are summarized in Figs 15 and 16, respectively. Our findings indicate that the increase of each stimulus effect increases both the cell elongation and CMI. Obviously, Figs 15 and 16 illustrate that the rate of changes in the cell elongation and CMI is greater in the direction of the electrotactic axis (*E*.Ω) than that of other cues ($\frac{\mu_{\text{ch}}+\mu_{\text{th}}}{\mu_{\text{mech}}}$), indicating dominant role of electrotaxis. Moreover, increasing the EF strength more than the saturation value does not remarkably affect the cell elongation and CMI. It should be mentioned that, generally, the greater the cell elongation and CMI the less cell random movement. The dominant role of the electrotaxis on cell directional movement is already discussed in the previous work in which a constant spherical cell shape was considered [67].

## Conclusions

In this study, our objective is to qualitatively characterize cell shape changes correlated with cell migration in the presence of multiple signals. Therefore, previously developed models of cell migration with constant spherical cell shape [67, 69] and mechanotactic effect on cell morphology [68] are here extended. The present 3D model is developed base on force equilibrium on cell body using finite element discrete methodology. This model allows predicting the cell behavior when it is surrounded by different micro-environmental cues. The results obtained here are qualitatively consistent with those of corresponding experimental works reported in the literature [13, 19, 20, 26, 96, 106].

In absence of external stimuli, the cell elongates along the stiffness gradient and migrates towards the surface of maximum stiffness. Although the cell may randomly extend different pseudopods, it retracts those pseudopods in subsequent steps and maintains its body in determinated distance from the surface of maximum elastic modulus, due to its unconstrained state. This is observed in the previous works of cell migration with a constant spherical shape as well [67, 69]. This causes a decrease in the cell elongation and CMI once the cell centroid is around IEP. The overall cell behavior and cell shape may be changed by activation of other signals in the cell environment. For instance, by adding chemotaxis and/or thermotaxis to the micro-environment, the maximum cell elongation and CMI increase and the location of the cell centroid moves towards the end of the substrate despite of the unconstrained boundary surface. As the cell migrate along chemical gradient, the cell elongates in gradient direction but when it is near the end of the substrate, the cell elongation and CMI decrease. Once the cell reaches the surface of maximum chemoattractant concentration, it extends pseudopods in the vertical direction of chemical gradient. Afterward, because the cell extends pseudopods in the vertical direction of chemical gradient, the cell elongation and CMI slightly increases, which is more obvious for greater chemical effective factor (Fig 12). The ultimate location of the cell centroid is sensitive to the chemotactic effective factors whereas employing of a higher chemoattractant effective factor causes that the cell centroid moves further to the end of the substrate. In other words, a greater chemoattractant effective factor dominates mechanotaxis signal and drives the cell towards the chemoattractant source. The cell movement to the end of the substrate is more critical in presence of electrotaxis. Since our study focuses on a typical cell migrating towards the cathode, EF significantly reorientates the cell towards the cathodal pole. This reorientation can be even considerably affected by increase of EF strength, in agreement with experimental observations [26].

So, generally, the stronger signal imposes a greater cell elongation and CMI that is because of directional cell polarisation towards the more effective stimulus. Because adding any new stimulus to the cell substrate will affect the cell polarization direction by increase of directional motility of the cell so that all signals directionally guide the cell towards the source of stimuli (warmer position, chemoattractant source, cathodal pole), diminishing the cell random movement (see Fig 8). In particular, in presence of the saturated EF there is a considerable increase in cell elongation and CMI due to exposing the cell to a greater electrostatic force. As a general remark, consistent with experimental observations, our findings indicate that electrotaxis effect is a dominant cue (see Figs 15 and 16). Because, for both the thermotactic and chemotactic signals, the variation of *μ*_th_ and *μ*_ch_ parameters has trivial effect on the magnitude of effective force (Equation 16), however it may considerably change the cell polarisation direction [67]. Therefore, changes of thermotaxis and chemotaxis slightly affect the magnitude of drag force in contrast to electrotaxis, which is an independent force from others, its magnitude can be directly controlled by the EF strength. Consequently, according to Equation 17 electrotaxis can affect both magnitude and direction of drag force. Taking together, this can clearly justify how electrotaxis is the most effective guiding mechanism of the cell elongation, CMI and the cell RI, which dominates other effective cues during cell motility, reported in many experimental works [6, 38, 110].

In summary, this study characterizes, for the first time, cell shape change accompanied with the cell migration change within 3D multi-signaling environments. We believe that it provides one step forward in computational methodology to simultaneously consider different features of cell behavior which are a concern in various biological processes. Although more sophisticated experimental works are required to calibrate quantitatively the present model, general aspects of the results discussed here are qualitatively consistent with documented experimental findings.

## Supporting Information

S1 Video Shape changes during cell migration within a substrate with a linear stiffness gradient. The substrate stiffness changes linearly in *x* direction from 1 kPa at *x* = 0 to 100 kPa at *x* = 400 *μ*m. At the beginning the cell is located in the soft region. The results demonstrate that the cell migrates in the direction of stiffness gradient and the cell centroid finally moves around an IEP located at *x* = 351 ± 5 *μ*m.
(AVI)

S2 Video Shape changes during cell migration within a substrate with conjugate linear stiffness and thermal gradients (*μ*_th_ = 0.2). It is assumed that there is a linear thermal gradient in x direction (as stiffness gradient) which changes from 36°C at x = 0 to 39°C at *x* = 400 *μ*m. At the beginning the cell is located near the surface with lower temperature. The results demonstrate that the cell migrates along the thermal gradient towards warmer region. Finally, the cell centroid moves around an IEP located at *x* = 359 ± 3 *μ*m. When the cell centroid is near the IEP the cell may send out and retract protrusions but it maintains the position around IEP.
(AVI)

S3 Video Shape changes during cell migration in presence of chemotaxis (*μ*_ch_ = 0.35) within a substrate with stiffness gradient. It is assumed that there is a chemoattractant substance with concentration of 5×10^−5^ M at *x* = 400 *μ*m, which creates a linear chemical gradient across *x* direction. At the beginning the cell is located near the surface of null chemoattractant substance. The results demonstrate that, the cell migrates along the chemical gradient towards the higher chemoattractant concentration. In this case, the cell centroid finally keeps moving around an IEP located at *x* = 368 ± 3 *μ*m. The ultimate position of IEP is sensitive to the chemical effective factor.
(AVI)

S4 Video Shape changes during cell migration in presence of chemotaxis (*μ*_ch_ = 0.40) within a substrate with stiffness gradient. It is assumed that there is a chemoattractant substance with concentration of 5×10^−5^ M at *x* = 400 *μ*m, which creates a linear chemical gradient across *x* direction. At the beginning the cell is located near the surface of null chemoattractant substance. The results demonstrate that, the cell migrates along the chemical gradient towards the higher chemoattractant concentration. For higher chemical effective factor, *μ*_ch_ = 0.4, the position of the IEP moves towards chemoattractant source to locate at at *x* = 374 ± 4 *μ*m.
(AVI)

S5 Video Shape changes during cell migration in presence of electrotaxis within a substrate with stiffness gradient. A cell is exposed to a dcEF (*E* = 10 mV/mm) where the anode is located at *x* = 0 and the cathode at *x* = 400 *μ*m. It is supposed that the cell is attracted to the cathode pole. At the beginning, the cell is placed near the anode and far from the cathode pole. The cell migrates along the dcEF towards the surface in which the cathode pole is located. Depending on EF strength, the ultimate location of the cell centroid will be different so that in this case (*E* = 10 mV/mm) the cell centroid keeps moving around an IEP located at *x* = 379 ± 3 *μ*m.
(AVI)

S6 Video Shape changes during cell migration in presence of electrotaxis within a substrate with stiffness gradient. A cell is exposed to a dcEF (*E* = 100 mV/mm) where the anode is located at *x* = 0 and the cathode at *x* = 400 *μ*m. It is supposed that the cell is attracted to the cathode pole. At the beginning, the cell is placed near the anode and far from the cathode pole. The cell migrates along the dcEF towards the surface in which the cathode pole is located. Depending on EF strength, the ultimate location of the cell centroid will be different so that in this case (*E* = 100 mV/mm) the cell centroid keeps moving around an IEP located at *x* = 383 ± 2 *μ*m.
(AVI)
